# Sagittal reconstruction of lumbosacral contiguous double-level spondylolytic spondylolisthesis: a comparison of double-level and single-level transforaminal lumbar interbody fusion

**DOI:** 10.1186/s13018-019-1197-7

**Published:** 2019-05-23

**Authors:** Chang-zhi Du, Song Li, Liang Xu, Qing-shuang Zhou, Ze-zhang Zhu, Xu Sun, Yong Qiu

**Affiliations:** 1 Department of Spine Surgery, Nanjing Drum Tower Hospital, Clinical College of Nanjing Medical University, Zhongshan Road 321, Nanjing, 210008 China; 2 Department of Spine Surgery, Nanjing Drum Tower Hospital, Medical School of Nanjing University, Nanjing, 210008 Jiangsu China

**Keywords:** Double-level spondylolysis, Isthmic spondylolisthesis, Spino-pelvic parameters, Transforaminal lumbar interbody fusion

## Abstract

**Background:**

Contiguous double-level lumbar spondylolytic spondylolisthesis is an extremely rare condition. There is a paucity of data of lumbosacral deformity and sagittal spino-pelvic malalignment among these patients. Moreover, the effect of transforaminal lumbar interbody fusion (TLIF) on sagittal realignment still remains largely unknown. The aim of the study is to investigate the reconstruction of sagittal alignment and the improvement of clinical outcomes after posterior instrumented double-level or single-level TLIF.

**Methods:**

From January 2010 to September 2018, the records of patients with contiguous L4/5 and L5/S1 double-level spondylolytic spondylolisthesis were retrospectively reviewed. Patients who had undergone double-level or single-level TLIF and a minimum of 2 years’ follow-up were included. The slippage parameters and spino-pelvic parameters were measured preoperatively, postoperatively, and at the latest follow-up.

**Results:**

A total of 58 patients (21 males and 37 females, mean age of 57.1 ± 6.9 years) were enrolled. Thirty-eight patients were treated with double-level TLIF and the remaining 20 with single-level TLIF (L4/5 in 14; L5/S1 in 6). After surgery, the spondylolisthesis was significantly reduced at both L4/5 and L5/S1 level (all *P* < 0.001). There was a significant reduction in pelvic tilt (*P* < 0.001) and a significant increase in sacral slope (*P* < 0.001). Significant increase in L4–S1 height (*P* < 0.001) and L4–S1 lordosis (*P* = 0.012) and decrease in L5 slope (*P* = 0.004) and L5 incidence (*P* = 0.001) were also observed. Compared to single-level TLIF, double-level TLIF increased L4–S1 height (*P* < 0.001) and L4–S1 lordosis (*P* < 0.001) and reduced L4-SVA (*P* = 0.007) and L5 incidence (*P* = 0.013) more obviously, and the sagittal balance was better corrected in double-level TLIF group (*P* = 0.006). Double-level TLIF group showed larger increase in VAS scores for low back pain. The incidence of implant-related complications was lower in the double-level group.

**Conclusion:**

Posterior short-segment instrumented TLIF can bring favorable radiographic and clinical outcomes in patients with lumbosacral contiguous double-level spondylolytic spondylolisthesis. Double-level TLIF is more efficient to improve L4–S1 height, regional lumbar lordosis, and global sagittal balance.

## Introduction

Lumbosacral contiguous double-level spondylolytic spondylolisthesis is an extremely rare condition with an incidence of 0.3–1.48% [1, 2]. Dysplastic weakness in the pars and repetitive overstress on the interarticularis are supposed to be the primary causes of double-level spondylolysis [3]. Secondary to spondylolysis, spondylolisthesis may arise in a time- and severity-dependent manner [4–6] and result in basic segmental kyphosis, short disc height, and even severe sagittal spino-pelvic imbalance [7]. Since the sagittal malalignment (more obvious forward trunk, more retroverted pelvis, and more loss of lumbar lordosis) caused by double-level degenerative spondylolisthesis differs from that by single-level degenerative spondylolisthesis [8, 9], it is therefore logically inferred that compared with the common single-level isthmic spondylolisthesis, some distinct spino-pelvic characteristics may also be demonstrated on lumbosacral contiguous double-level spondylolytic spondylolisthesis.

Spinal instrumented fusion surgery serves as an optimal treatment for severe progressive spondylolisthesis, with or without obvious persistent radicular pain or neurological claudication [1, 10, 11]. Previous studies have highlighted the importance of spondylolisthesis reduction and restoration of sagittal lumbosacral alignment [12]. Transforaminal lumbar interbody fusion (TLIF) is preferable in treating spondylolisthesis [13, 14] for its powerful ability to reduce anterolisthesis, restore disc height, and improve clinical outcomes [15, 16]. In clinical practice, both double-level TLIF and selective single-level TLIF are employed for contiguous double-level spondylolytic spondylolisthesis. However, the radiographic and clinical outcomes of posterior instrumented TLIF have never been investigated, and it remains unknown whether double-level TLIF produced better results than single-level TLIF.

The present study reviewed the medical records of a consecutive series of patients with lumbosacral contiguous double-level spondylolytic spondylolisthesis who had been treated with posterior instrumentation and TLIF. The hypotheses were as follows: (1) posterior instrumentation and TLIF were effective to correct the regional lumbosacral malalignment and sagittal spino-pelvic profile and (2) double-level TLIF was superior to selective single-level TLIF in sagittal alignment reconstruction.

## Materials and methods

### Study population

With the approval from the Hospital Ethics Committee, we retrospectively reviewed the consecutive patients with double-level lumbar spondylolytic spondylolisthesis from January 2010 to September 2018. Isthmic spondylolysis was defined as the bilateral interarticularis defect on CT scans, and spondylolisthesis as the cephalic vertebra anteriorly slipping over the caudal vertebra by ≥ 5 mm in the sagittal plane of standardized standing lateral X-ray [17, 18]. Patients meeting the following criteria were included: contiguous L4 and L5 double-level spondylolytic spondylolisthesis; progressive persistent low back pain, with or without radicular pain and neurological claudication (unsatisfied with the outcomes of conservative treatments); surgically treated with L4–S1 instrumented fusion and TLIF; complete radiographs of spine and other clinical data; and follow-up of more than 2 years. The exclusion criteria were as follows: lumbar degenerative spondylolisthesis, coronal curve over 10°, history of spinal trauma or surgery, and disorders of the pelvis or lower extremities. Finally, a total of 58 patients (21 males and 37 females), with the average age at surgery of 57.1 ± 6.9 years, were enrolled in the current study.

### Surgical intervention

All patients were treated with posterior pedicle screw fixation from L4 to S1 by the senior surgeons. TLIF procedures were generally performed at both spondylolytic levels (L4/5 and L5/S1 levels). Whereas, for patients with poor economic condition, posterior instrumentation without TLIF was performed at the non-dominant spondylolisthesis level with a less degenerated disc and no evident of foraminal or central canal stenosis [19, 20]. Instead, intertransverse bone graft was carried out for fusion. Therefore, the patients were assigned to two sub-groups, double-level TLIF group (TLIF performed at both L4/L5 and L5/S1 levels) and single-level TLIF group (TLIF performed at L4/L5 or L5/S1 level).

After general anesthesia, the patient was placed in a prone position. A midline incision was opened in a conventional way, then the fascia and paraspinal muscles retracted with self-retaining retractors. The pedicle screws were inserted using the freehand technique. A bilateral facetectomy at the level of fusion and standard laminectomy was carried out at the level requiring decompression. TLIF procedures were performed at the side with dominant symptoms [12]. After thorough discectomy and endplate preparation, the rods were placed bilaterally to reduce forward slippage. A PEEK cage with auto-graft was inserted into the disc space, then the pedicle screw compressed to rebuild segmental lordosis. The cage size was mainly chosen empirically and set according to the height of the disc space.

### Radiographic measurements

**Fig. 1:**
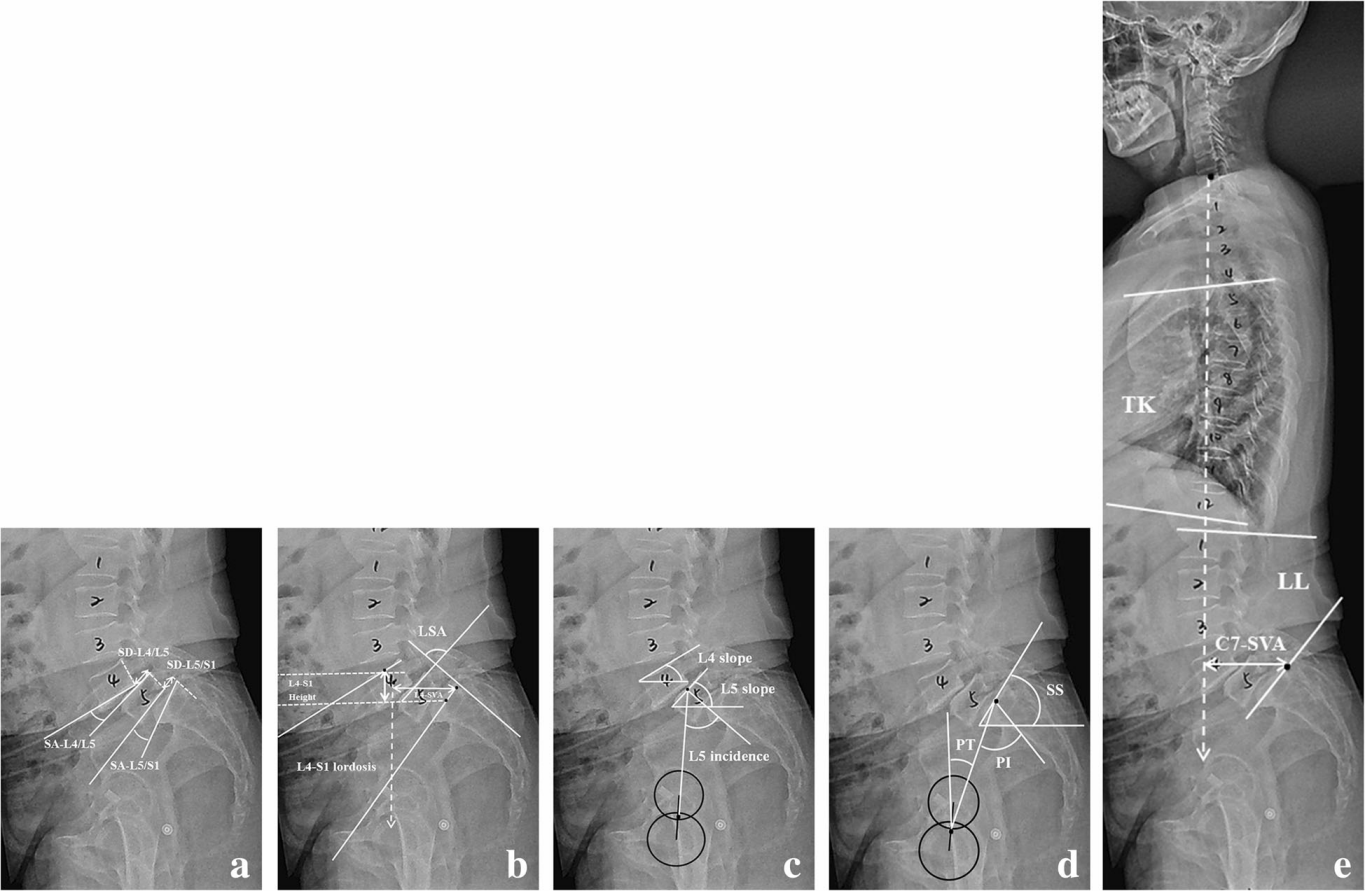
The measurement of radiographic parameters. **a** Slippage parameters. **b**, **c** Deformity parameters. **d** Pelvic parameters. **e** Sagittal spinal parameters

All radiographic parameters were measured on a standardized standing lateral full-length X-ray of the whole spine by an experienced spine surgeon using Surgimap Spine Software (Surgimap, New York, USA). The measurement was conducted twice, and the average was used for each data. All radiographic parameters were measured preoperatively, postoperatively (within 3 months), and at the latest follow-up (Fig. 1).

The slippage parameters included slip distance (SD), slip angle (SA), slip percentage (SP). The definitions of these parameters are as follows: SD, the interval between two extended lines of the posterior aspect of upper and lower vertebral body; SA, the angle between the inferior endplate of upper vertebrae and the superior endplate of lower vertebrae; and SP, the percentage of SD to the superior endplate of the caudal vertebrae based on the Meyerding classification [21]. The reduction rate of slippage was calculated by percentage of preoperative SP minus postoperative SP to preoperative SP.

To identify the regional lumbosacral malalignment, L4–S1 height, L4–S1 lordosis, lumbosacral angle (LSA), L4 slope, L5 slope, and L5 incidence were measured. Their definitions were as follows: L4–S1 height, the vertical distance between the superior endplate midpoint of L4 and that of S1; L4–S1 lordosis, the angle between the superior endplate of L4 and the superior endplate of S1; L4 sagittal vertical axis (L4-SVA), the distance between the L4 plumb line and the posterosuperior corner of S1; LSA, the angle between the tangent posterior edge of S1 and the superior endplate of L5; L4 slope, the angle between the superior endplate of L4 and the horizontal plane; L5 slope, the angle between the superior endplate of L5 and the horizontal plane; and L5 incidence, the angle between the perpendicular bisector of L5 superior endplate and the line connecting midpoints of L5 superior endplate and the center of femoral head. The outcome of lumbosacral reconstruction was evaluated with these parameters after surgery.

The pelvic parameters included pelvic incidence (PI), pelvic tilt (PT), and sacral slope (SS); spinal parameters included L4–S1 lordosis, lumbar lordosis (LL), thoracic kyphosis (TK), and C7 sagittal vertical axis (C7-SVA). The sagittal balance was determined according to the horizontal distance from the C7 plumb line to the posterior superior corner of S1 (C7-SVA), and imbalance was considered to be present if the distance surpassed 40 mm. Kyphotic angle was regarded as negative and lordotic angle as positive. In addition, cage subsidence (the intervertebral cage embedded into the adjacent vertebral endplate by > 2 mm) and cage migration (any offset of ≥ 1 mm in the axial plane) were recorded.

### Data collection and clinical evaluation

For each patient, the demographic and perioperative data was reviewed, including age, gender, body mass index (BMI), bone mineral density (BMD), smoking status, duration of symptoms, follow-up period, duration of surgery, intraoperative blood los,s and hospital stay.

The patient-reported outcome was prospectively collected before and after surgery during the hospital stay. After hospital discharge, a series of questionnaires were completed through a face-to-face interview at their routine outpatient visits. In addition, telephone interviews with the patients were also made at least once a year. Life quality and disability severity were evaluated using the Oswestry Disability Index (ODI), the Short Form-36 (SF-36) Physical Health, and the visual analog scale (VAS). Unplanned surgeries or implant-related complications were recorded.

### Statistical analysis

Data was analyzed using SPSS 22.0 (SPSS Inc, Chicago, IL). All values were presented as mean ± standard deviation. Student’s *t* test was used to compare the difference in each radiographic or clinical parameter between the double-level TLIF group and single-level TLIF group. In each group, the radiographic outcomes at different timepoints (preoperatively, postoperatively, and at the latest follow-up) were compared with the use of paired *t* test. *P*<0.05 was considered statistically significant.

## Results

### Demographic data and preoperative parameters

**Fig. 2:**
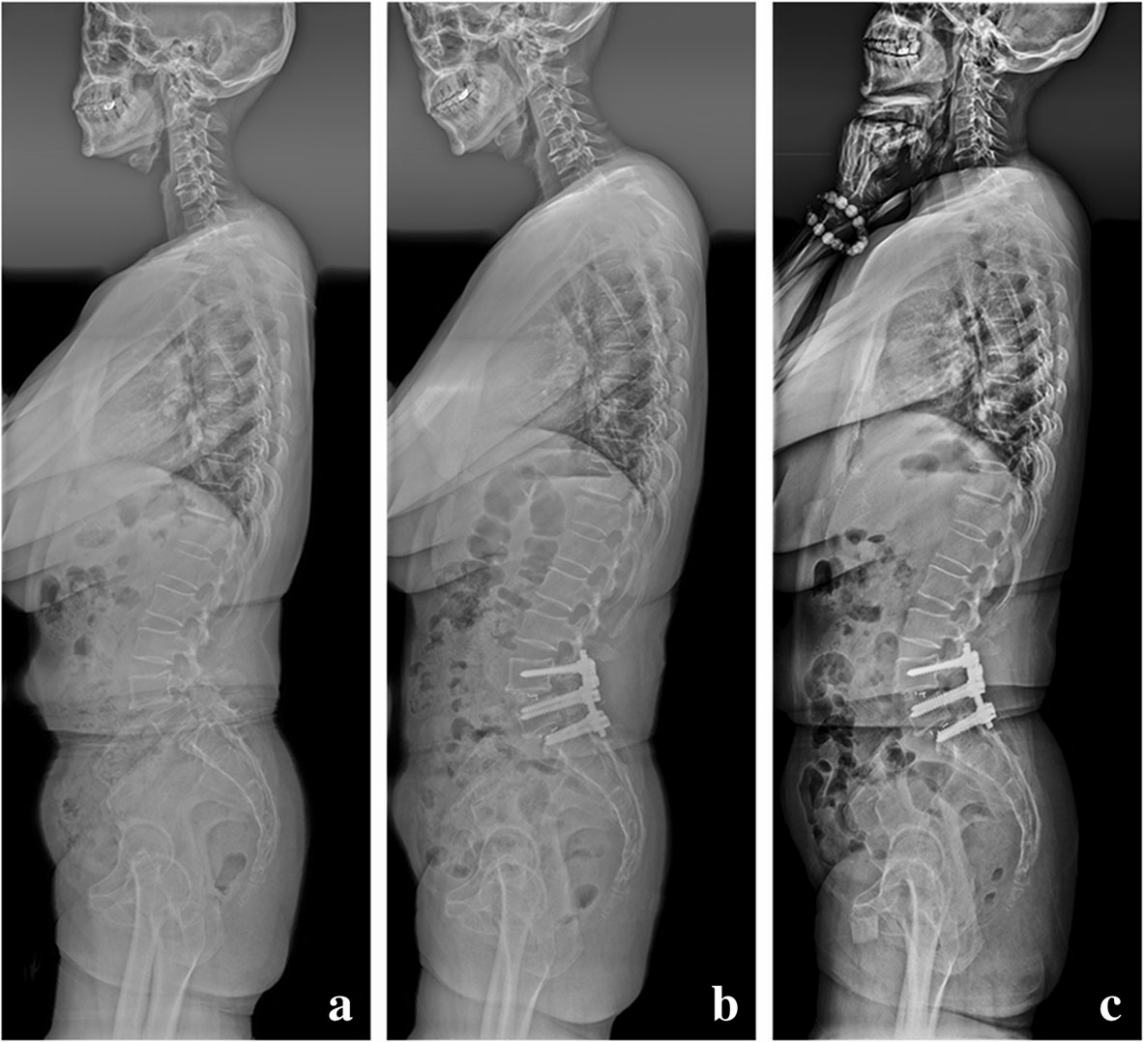
54-year-old female with L4/5 and L5/S1 double-level spondylolytic spondylolisthesis. **a** The preoperative radiograph showed a distinct lumbosacral deformity (L4–S1 height = 51.3 mm, L4-SVA = 34.3 mm) and sagittal imbalance (L4–S1 lordosis/LL = 44.5%, C7-SVA = 43.8 mm). **b** Postoperative radiograph following L4/L5 and L5/S1 dual-level TLIF and posterior instrumentation from L4 to S1 showed a good restoration of L4–S1 region (L4–S1 height = 68.5 mm, L4-SVA = 18.7 mm), an optimal redistribution of lumbar lordosis (L4–S1 lordosis/LL = 67.5%), and a harmonic global sagittal balance (C7-SVA = 8.4 mm). **c** Radiograph obtained at 4 years after surgery revealed that both lumbosacral region and global sagittal alignment maintained well.

**Fig. 3:**
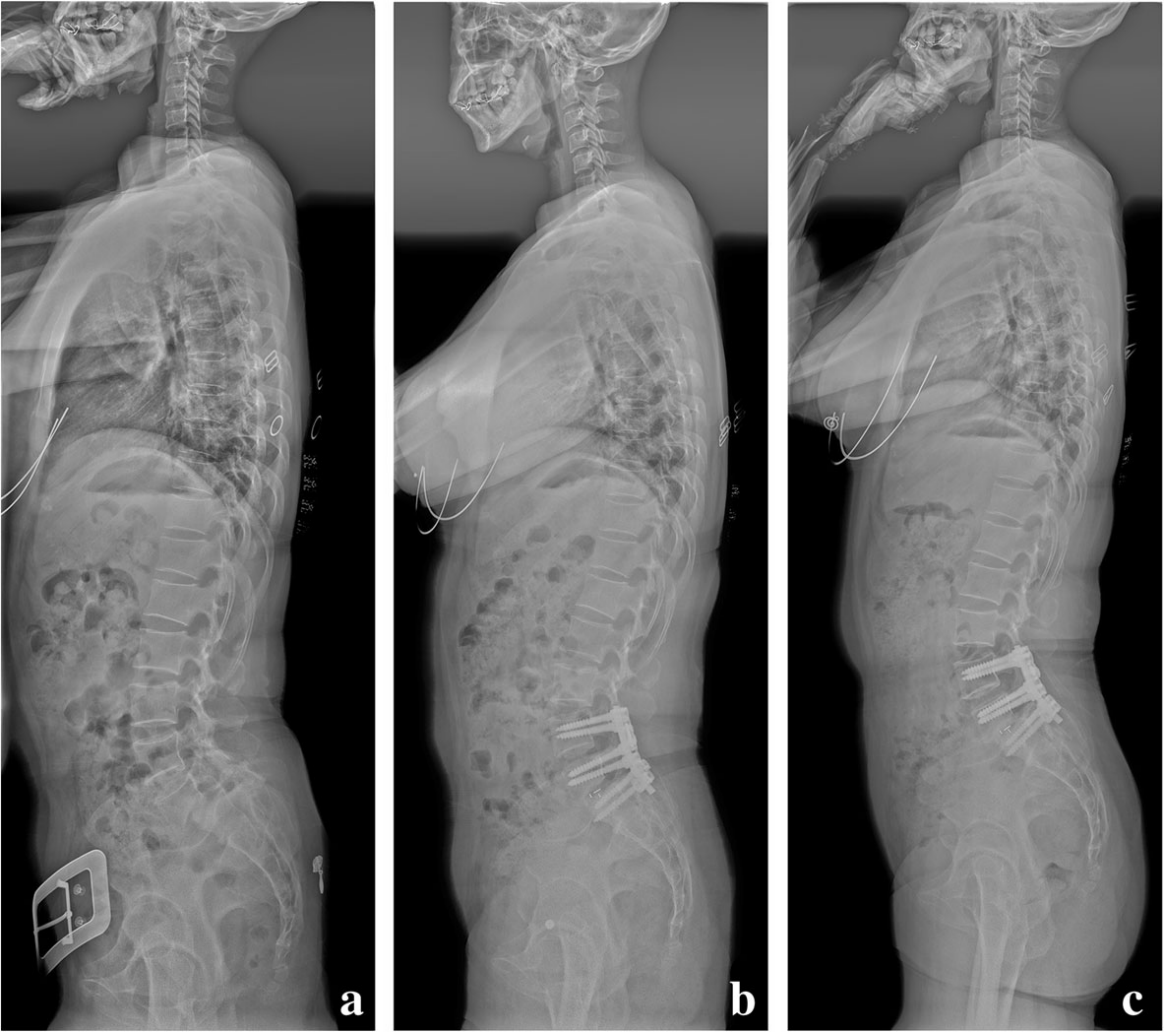
60-year-old female with L4/5 and L5/S1 double-level spondylolytic spondylolisthesis. **a** The preoperative radiograph demonstrated an obvious lumbosacral deformity (L4–S1 height = 56.9 mm, L4-SVA = 32.5 mm) and sagittal spinal imbalance (L4–S1 lordosis/LL = 48.3%, C7-SVA = 38.2 mm). **b** Postoperative radiograph after L5/S1 single-level TLIF and posterior instrumentation from L4 to S1 demonstrated an obviously corrected of segmental and global sagittal alignment (L4–S1 height = 64.1 mm, L4-SVA = 22.3 mm, L4–S1 lordosis/LL = 58.6%, C7-SVA = 12.4 mm). **c** Radiograph at postoperative 3 years revealed a good sagittal spino-pelvic alignment.

Table 1 summarizes the general information of the cohort. There were 38 cases (65.5%) in the double-level TLIF group (Fig. 2) and 20 cases in the single-level TLIF group (Fig. 3) (L4/5 TLIF, 14 cases, 24.1%; L5/S1 TLIF, 6 cases, 10.4%). The double-level TLIF group was composed of 15 males and 23 females with the mean age of 54.4 ± 9.4 years), while the single-level TLIF group consisted of 6 males and 14 females with the age of 58.2 ± 5.7 years. There were no significant differences between the two groups in age, gender, smokers, BMI, T score of BMD, and the duration of symptoms. In terms of the preoperative radiographic parameters, the single-level TLIF group had insignificantly higher disc height and lower disc Pfirrmann grade at both L4–5 and L5–S1 level compared to the double-level TLIF group (*P* > 0.05, respectively). In addition, there were also no significant differences between the two groups for slip percent and Modic change between the two groups (*P* > 0.05, respectively).

**Table 1:** Demographic data and preoperative parameters

	In total	Double-level TLIF group	Single-level TLIF group	*P* value
Number of patients	58	38	20	
Gender, *n* (%)				0.415
Male	21 (36.2%)	15 (39.5%)	6 (30.0%)	
Female	37 (63.8%)	23 (60.5%)	14 (70.0%)	
Age at surgery, year	57.1 ± 6.9	54.4 ± 9.4	58.2 ± 5.7	0.104
Smokers, *n* (%)	10 (17.2%)	7 (18.4%)	3 (15.0%)	0.327
BMI, kg/m^2^	26.1 ± 4.2	26.8 ± 5.2	24.7 ± 3.6	0.112
T score of BMD	− 1.3 ± 1.1	− 1.1 ± 1.4	− 1.5 ± 0.9	0.252
The duration of symptoms, month	18.5 ± 7.6 (6–46)	19.7 ± 9.3 (10–46)	18.1 ± 6.6 (6–39)	0.497
Follow-up period, month	31.8 ± 10.7 (24–73)	34.2 ± 11.8 (24–73)	30.5 ± 9.3 (26–65)	0.229
L4–L5 level				
Slip percent, %	21.6 ± 10.4%	22.3 ± 13.4%	(18.1 ± 15.8)%	0.290
Modic change, *n* (%)	13 (22.4%)	9 (23.6%)	4 (20.0%)	0.742
Disc height, mm	10.9 ± 2.6	10.6 ± 2.3	11.7 ± 2.8	0.114
Disc Pfirrmann grade	3.2 ± 1.7	3.6 ± 1.6	2.8 ± 2.1	0.110
L5–S1 level				
Slip percent	17.1 ± 12.3%	20.1 ± 14.6%	15.4 ± 13.8%	0.240
Modic change incidence	16 (27.6%)	10 (26.3%)	6 (30.0%)	0.683
Disc height, mm	9.3 ± 3.1	8.9 ± 2.8	10.2 ± 3.7	0.138
Disc Pfirrmann grade	3.1 ± 1.8	3.2 ± 1.4	2.5 ± 2.3	0.155

*BMI* body mass index. *P* value for the comparison between double-level TLIF group and single-level TLIF group

### Perioperative parameters

The summary of perioperative parameters is presented in Table 2. The surgical duration and intraoperative blood loss were all more in the double-level TLIF group (298.6 ± 29.6 vs. 246.5 ± 26.5 min, *P* < 0.001; 802.2 ± 74.6 vs. 684.5 ± 60.5 ml, *P* < 0.001, respectively). The mean hospital stay was longer in the double-level TLIF group than in the single-level TLIF group (16.8 ± 3.6 vs. 15.5 ± 4.8 days); however, this difference was not statistically significant (*P* = 0.249).

**Table 2:** Perioperative parameters of both groups

	In total	Double-level TLIF group	Single-level TLIF group	*P* value
Surgical duration, minute	278.2 ± 35.8	298.6 ± 29.6	246.5 ± 26.5	< 0.001
Intraoperative blood loss, ml	741.3 ± 96.2	802.2 ± 74.6	684.5 ± 60.5	< 0.001
Hospital stay, day	16.3 ± 4.2	16.8 ± 3.6	15.5 ± 4.8	0.249

### Slippage parameters

The mean postoperative follow-up period was 34.2 ± 11.8 months (ranging from 24 to 73 months) in the double-level TLIF group and 30.5 ± 9.3 months (ranging from 26 to 65 months) in the single-level TLIF group. As shown in Table 3, after surgery, the spondylolisthesis at L4/5 and L5/S1 was reduced by 74.6% ± 12.4% and 58.3% ± 9.6%, respectively. By the latest follow-up, solid fusion was achieved in both L4/5 and L5/S1levels.

**Table 3:** Slippage parameters at different timepoints

	L4/5 level			L5/S1 level		
	SD (mm)	SA (°)	SP	SD (mm)	SA (°)	SP
Pre-OP	8.0 ± 4.1	− 7.9 ± 6.4	21.6 ± 10.4%	6.2 ± 4.1	− 5.9 ± 6.8	17.1 ± 12.3%
Post-OP	2.9 ± 2.0*	− 9.1 ± 4.3	6.1 ± 3.9%*	3.0 ± 1.7*	− 9.6 ± 4.5*	7.6 ± 4.8%*
Latest FU	2.6 ± 1.8	− 8.2 ± 4.2	6.3 ± 4.4%	3.1 ± 1.9	− 9.1 ± 5.1	7.9 ± 5.0%

*SD* slip distance, *SA* slip angle, *SP* slip percent, *OP* operation, *FU* follow-up, *vs* versus

^*^Comparison between preoperation and postoperation

### Radiographic evaluation of lumbosacral malalignment and sagittal spino-pelvic parameters

As shown in Table 4, the segmental lumbosacral deformity and sagittal spino-pelvic parameters were significantly improved after surgery. In particular, the L4–S1 height increased from 52.7 ± 12.5 to 69.2 ± 11.0 mm (*P* < 0.05), and the L4-SVA length decreased from 32.0 ± 13.7 to 19.7 ± 16.2 mm (*P* < 0.05). In addition, both L4–S1 lordosis and LSA increased significantly, and both L5 slope and L5 incidence decreased significantly after surgery (*P* < 0.05, respectively). The ratio of L4–S1 to LL increased from 46.8 ± 18.3% to 59.8 ± 23.1%, and that of C7 to SVA decreased from 31.3 ± 34.2 mm to − 1.7 ± 19.2 mm (*P* < 0.05, respectively).

**Table 4:** Lumbosacral deformity parameters and sagittal spino-pelvic parameters

	In total			Double-level TLIF group				Single-level TLIF group				*P* value (change)
	Pre-OP	Post-OP	Latest FU	Pre-OP	Post-OP	Latest FU	Change	Pre-OP	Post-OP	Latest FU	Change	
Lumbosacral deformity parameters												
L4–S1 height, mm	52.7 ± 12.5	73.4 ± 14.3*	69.2 ± 11.0	50.1 ± 11.4	75.2 ± 13.6*	72.8 ± 10.6	22.7 ± 7.1	55.1 ± 10.5	71.8 ± 9.3*^§^	66.0 ± 8.2	10.9 ± 4.8	< 0.001
L4–S1 lordosis, °	26.2 ± 12.9	34.8 ± 14.6*	32.8 ± 15.1	23.6 ± 10.9	38.5 ± 11.3*	35.4 ± 12.1	11.8 ± 9.1	27.5 ± 9.3	31.6 ± 11.4	30.5 ± 14.3	3.0 ± 6.2	< 0.001
L4-SVA, mm	32.0 ± 13.7	19.7 ± 16.2*	22.8 ± 14.8	33.2 ± 10.4	17.4 ± 12.3*	20.8 ± 13.5	− 12.4 ± 8.2	31.1 ± 12.8	21.7 ± 14.3*	24.6 ± 12.6	− 6.5 ± 6.4	0.007
LSA, °	106.8 ± 15.5	114.3 ± 17.1*	116.2 ± 16.9	104.2 ± 14.2	116.2 ± 16.3*	118.3 ± 15.5	14.1 ± 11.9	109.1 ± 13.6	112.5 ± 13.8	114.4 ± 12.7	5.3 ± 9.5	0.006
L4 slope, °	10.1 ± 12.7	5.9 ± 14.2	6.6 ± 9.8	12.2 ± 12.3	7.4 ± 6.1*	8.3 ± 7.4	− 3.9 ± 7.6	8.2 ± 11.4	4.6 ± 12.1	5.1 ± 9.2	− 3.1 ± 5.6	0.680
L5 slope, °	24.5 ± 14.4	17.6 ± 14.8*	17.7 ± 10.2	27.1 ± 13.3	17.4 ± 12.3*	19.2 ± 8.9	− 8.1 ± 4.2	22.1 ± 9.3	17.8 ± 10.3	16.4 ± 8.1	− 5.6 ± 3.1	0.022
L5 incidence, °	36.1 ± 15.3	26.9 ± 16.9*	28.0 ± 11.6	39.7 ± 12.2	28.2 ± 14.3*	30.2 ± 9.7	− 9.5 ± 4.4	32.7 ± 11.6	25.7 ± 12.8*	26.1 ± 9.3	− 6.6 ± 3.5	0.013
Sagittal spino-pelvic parameters												
PI, °	63.0 ± 13.4	63.9 ± 14.5	64.1 ± 12.7	66.2 ± 12.6	67.5 ± 13.7	67.3 ± 12.1	1.1 ± 1.3	60.2 ± 11.3	60.8 ± 14.2	61.4 ± 10.1	1.2 ± 1.5	0.792
PT, °	30.5 ± 11.6	19.0 ± 13.5*	22.1 ± 9.4	31.9 ± 10.4	18.4 ± 12.3*	21.4 ± 8.9	− 9.8 ± 6.7	29.3 ± 10.8	19.6 ± 12.8*	22.8 ± 7.8	− 6.5 ± 6.3	0.074
SS, °	32.4 ± 11.4	45.3 ± 11.1*	42.5 ± 9.6	34.8 ± 6.8	47.9 ± 10.5*	44.2 ± 8.6	9.4 ± 6.3	31.9 ± 9.4	43.6 ± 8.3*	41.7 ± 7.9	9.8 ± 4.3	0.800
TK, °	16.5 ± 10.4	27.4 ± 13.2*	24.8 ± 11.8	14.1 ± 8.1	25.2 ± 10.2*	22.3 ± 9.8	8.2 ± 6.4	18.6 ± 8.7	29.4 ± 9.3*	27.1 ± 5.2	8.5 ± 8.7	0.881
LL, °	54.7 ± 13.1	55.2 ± 17.1	56.4 ± 14.6	52.4 ± 10.6	56.2 ± 14.2	57.6 ± 13.1	5.2 ± 8.1	56.7 ± 12.1	54.3 ± 15.2	55.4 ± 13.7	− 1.3 ± 10.1	0.010
L4–S1 lordosis/LL, %	46.8 ± 18.3	63.1 ± 16.7*	59.8 ± 23.1	43.7 ± 15.3	68.4 ± 14.2*	65.1 ± 21.3	21.4 ± 10.3	49.7 ± 16.0	58.3 ± 12.4	55.2 ± 16.5	5.5 ± 7.5	< 0.001
C7-SVA, mm	31.3 ± 34.2	10.6 ± 23.6*^§^	− 1.7 ± 19.2	37.8 ± 32.2	8.4 ± 21.4*^§^	− 9.6 ± 17.2	− 47.4 ± 21.9	26.4 ± 22.7	12.6 ± 16.8*	5.4 ± 14.8	− 21.0 ± 18.1	< 0.001

*LSA* lumbosacral angle, *PI* pelvic incidence, *PT* pelvic tilt, *SS* sacral slope, *TK* thoracic kyphosis, *LL* lumbar lordosis, *L4–S1 lordosis/LL* the ratio of L4–S1 lordosis to LL, *SVA* sagittal vertical axis, *OP* operation, *FU* follow-up, *vs* for versus. Change = the value of latest FU − the value of pre-OP

*Comparison between preoperation and postoperation

§Comparison between postoperation and latest follow-up

Compared with the single-level TLIF group, the double-level TLIF group showed a larger increase in L4–S1 height, L4–S1 lordosis, and the ratio of L4–S1 lordosis to LL and LSA and a larger decrease in L4-SVA after surgery (all *P* < 0.05). At the final follow-up, the double-level TLIF group also showed a larger decrease in L5 slope and L5 incidence (*P* < 0.05). Despite a greater preoperative C7-SVA, the sagittal balance was better corrected in the double-level TLIF group than in the single-level TLIF group (*P* = 0.006).

### Clinical outcomes

As shown in Table 5, there was a significant decrease in low back and leg VAS scores and ODI scores and a significant increase in SF-36 scores after surgery (all *P* < 0.05). Besides, the single-level TLIF group showed less favorable improvement in VAS scores of low back pain compared with the double-level TLIF group (*P* = 0.010). However, there was no significant difference in terms of ODI scores, SF-36 scores, and VAS scores of leg pain between the double-level TLIF group and single-level TLIF group (all *P* > 0.05).

**Table 5:** Patient-reported outcomes

	In total		Double-level TLIF group			Single-level TLIF group (*N* = 20)			*P* value (change)
	Pre-OP	Latest FU	Pre-OP	Latest FU	Change	Pre-OP	Latest FU	Change	
ODI	54.2 ± 22.3	30.1 ± 21.4*	56.3 ± 21.3	31.7 ± 16.4*	− 24.6 ± 12.5	52.4 ± 17.6	33.2 ± 19.3*	− 19.2 ± 15.6	0.157
SF-36 Physical Health	33.6 ± 9.4	44.9 ± 7.6*	32.6 ± 7.8	45.7 ± 6.3*	13.0 ± 6.9	34.5 ± 5.7	44.3 ± 6.7*	9.8 ± 5.1	0.073
VAS of low back pain	6.7 ± 3.7	3.0 ± 3.2*	7.2 ± 3.5	2.9 ± 1.7*	− 4.3 ± 1.6	6.3 ± 3.1	3.2 ± 2.6*	− 3.1 ± 1.7	0.010
VAS of leg pain	7.9 ± 3.4	2.6 ± 2.5*	8.1 ± 2.2	2.4 ± 1.6*	− 5.7 ± 2.4	7.8 ± 2.7	2.9 ± 1.9*	− 4.9 ± 2.5	0.239

*ODI* Oswestry Disability Index, *SF-36* Short Form-36, *VAS* visual analog scale, *OP* operation, *FU* follow-up

Change = the value of latest FU − the value of pre-OP

*Comparison between preoperation and latest follow-up

### Complications

The incidence of postoperative complications was similar between the two groups. In the double-level TLIF group, delayed wound healing due to surgical site infection was found in one patient and cage subsidence in one patient. In the single-level TLIF group, symptomatic adjacent segment disease occurred to one patient and implant failure due to pseudoarthrosis occurred to one patient. All the abovementioned complications were treated conservatively. None had undergone a revision surgery.

## Discussion

The present study reported a total of 58 patients with contiguous L4–S1 double-level spondylolytic spondylolisthesis. To our knowledge, this is the first comprehensive study, with the largest sample so far [1, 22–26], that investigates spondylolisthesis reduction and sagittal lumbosacral reconstruction with posterior L4–S1 instrumented fusion and TLIF. In this study, spondylolisthesis always occurred with severe regional lumbosacral deformity and resultant sagittal malalignment. After surgery, segmental and global sagittal profiles were restored. Besides, the double-level TLIF brought with better sagittal profile and clinical outcomes and less implant-related complications than the single-level TLIF.

Contiguous L4 and L5 spondylolytic spondylolisthesis leads to shorter disc height and more obvious forward displacement of L4/5 and L5/S1 altogether than commonly seen single-level isthmic spondylolisthesis. These segmental deformities might be mainly responsible for spino-pelvic sagittal malalignment. Our study, with most patients of grade I spondylolisthesis [27], further highlighted the critical importance of lumbosacral deformity correction in sagittal reconstruction. Park et al. [16] reported that restoring disc space was the key to achieve a favorable surgical outcome. Our results confirmed this finding. Moreover, with the realignment of both L4 and L5 vertebrae after instrumentation from L4 to S1, we observed a significant increase in L4–S1 lordosis and its ratio to LL and a decrease in L4-SVA. The effect of TLIF on regional deformity correction has also been verified as increasing lumbosacral lordosis and significant improvements in sacral inclination and disc height [15]. Theoretically, the sagittal balance can be regained through reconstructing segmental lumbosacral alignment. The results of our study showed a significant reduction in C7-SVA and increase in TK after surgery. Therefore, posterior instrumentation and TLIF, with its ability to reduce spondylolisthesis and lumbosacral deformity, can rebuild a harmonious sagittal spino-pelvic alignment in patients with contiguous double-level lumbar spondylolytic spondylolisthesis.

The present study demonstrated that double-level TLIF had a distinct advantage over selective single-level TLIF in correcting regional lumbosacral malalignment. It is supposed that TLIF at more levels might bring larger increment in L4–S1 height. Kepler et al. [28] described the magnitude of relordosis was 3.6–5.5° per segment after TLIF, indicating that double-level TLIF may increase lumbosacral lordosis through reconstructing L4/5 and L5/S1 levels simultaneously. Janik et al. [29] argued that the best representation of regional lordosis from L4 to S1 was two third of lumbar lordosis. Our results showed that the lumbar lordosis was normalized after double-level TLIF, with a ratio of L4–S1 lordosis to LL up to 65.1%. These changes may be attributed to the restoration of L5 vertebra in the lumbosacral region, as evidenced by the significant decrease in both L5 slope and L5 incidence after double-level TLIF. In recent years, the above two parameters of L5 vertebra have also been used to evaluate the successful restoration of the spino-pelvic sagittal alignment [30].

Regaining global sagittal balance is a surgical goal for symptomatic progressive spondylolisthesis. Sagittal imbalance with a large C7-SVA is always found before surgery in patients with contiguous L4–S1 double-level spondylolytic spondylolisthesis. Since C7-SVA correlates negatively with patient-reported outcomes, reducing C7-SVA after surgery is of crucial value to improve clinical outcomes [31]. In the present study, C7-SVA decreased significantly after posterior instrumentation and TLIF. In particular, double-level TLIF reaped better sagittal rebalance, which might be largely attributed to the excellent lumbosacral sagittal reconstruction. In addition, the surgical potential superiority of TLIF, with more direct spinal canal decompression and greater change of foramen area after meticulous foraminotomy [32], may also have positive effects to some extent.

This study, with the largest sample so far, has thoroughly examined the effectiveness of surgical treatment for contiguous L4–S1 double-level spondylolytic spondylolisthesis. Posterior instrumented TLIF is a classic procedure for spondylolisthesis. Insertion of more artificial cages incurs extra cost for the TLIF (approximately $2000 for the PEEK cage in China). In our study, for patients with poor economic condition, posterior instrumentation without TLIF was performed at the negligible spondylolisthesis level with a good disc height and morphology, and intertransverse bone graft was carried out for fusion instead. The clinical outcomes were similar between selective single-level TLIF and double-level TLIF. Our study indicates that selective single-level TLIF was also a feasible and effective treatment for patients with poor economic condition.

However, it has still several limitations. Firstly, our patients were a specifically selected population of contiguous L4 and L5 double-level spondylolytic spondylolisthesis and L4–S1 instrumentation, and patients with L3 spondylolytic defects or posterior fixation extending to L3 or upper were excluded. Secondly, this study did not compare the effectiveness of TLIF with other interbody fusion techniques, and the surgical advantages of TLIF still need to be further investigated by multicenter, long-term follow-up.

## Conclusion

Posterior L4–S1 instrumented TLIF can reduce spondylolisthesis, restore the sagittal lumbosacral alignment, and improve the clinical outcomes of patients with contiguous double-level lumbar spondylolytic spondylolisthesis. Double-level TLIF can produce a better restoration of L4–S1 height, an optimal redistribution of the lumbar lordosis, and a more harmonious realignment of global sagittal balance.

## Abbreviations

LL: Lumbar lordosis
LSA: Lumbosacral angle
ODI: Oswestry Disability Index
PI: Pelvic incidence
PT: Pelvic tilt
SA: Slip angle
SD: Slip distance
SF-36: Short Form-36
SP: Slip percentage
SS: Sacral slope
SVA: Sagittal vertical axis
TK: Thoracic kyphosis
TLIF: Transforaminal lumbar interbody fusion
VAS: Visual analog scale
